# Occurrence and Phenotypic Characteristics of Methicillin-Resistant *Staphylococcus aureus* (MRSA) in Emergency Medical Service Ambulances as a Potential Threat to Medical Staff and Patients

**DOI:** 10.3390/jcm13237160

**Published:** 2024-11-26

**Authors:** Piotr Konrad Leszczyński, Aleksandra Olędzka, Kamila Wierzchowska, Aneta Frankowska-Maciejewska, Krzysztof Marek Mitura, Daniel Celinski

**Affiliations:** 1 Faculty of Medical and Health Sciences, University of Siedlce, 08-110 Siedlce, Poland; 2 Independent Public Health Care Center RM-MEDITRANS Emergency Station and Sanitary Transport in Siedlce, 08-110 Siedlce, Poland; 3 Department of Emergency Medical Service, Medical University of Warsaw, 02-091 Warsaw, Poland

**Keywords:** ambulance, antibiotics, emergency medical service, infection, MRSA (methicillin-resistant *Staphylococcus aureus*), MSSA (methicillin-sensitive *Staphylococcus aureus*)

## Abstract

**Introduction:** An ambulance used by an emergency medical service team is the workplace of specialised medical personnel, providing daily transportation for patients in life-threatening conditions, from all walks of life, with numerous diseases and injuries. MRSA (methicillin-resistant *Staphylococcus aureus*) strains are classified as Gram-positive cocci, characterised primarily by their multidrug resistance. Infections caused by *S. aureus* have a low treatment success rate and are associated with persistent carrier state. This study aimed to isolate MRSA and MSSA (methicillin-sensitive *Staphylococcus aureus*) in the emergency vehicle and determine drug resistance of these isolates. **Materials and Methods:** This study involved an ambulance vehicle operated in central Poland. A total of 39 swabs were taken and evaluated from inside the ambulance on permanent duty. The isolates were analysed using catalase and coagulase assays, Gram staining, culturing on Chapman medium, growth evaluation on agar with 5% sheep blood, and assessing the strains’ sensitivities to selected antibiotics. Material was collected from 13 designated points located in the medical compartment and driver’s cabin. **Results:** *S. aureus* bacteria were detected in 51.28% of the samples, 40% of which were MRSA strains. Despite the application of high disinfection standards for the interior of the ambulance, it was not possible to kill all *S. aureus* strains, which may be because the pathogens in question produce a biofilm that effectively allows them to survive on various surfaces, including those disinfected. Almost 100% of the MRSA isolates were resistant to antibiotics from the β-lactam group (penicillin, ticarcillin, cefotaxime, and cefoxitin), the macrolide group (erythromycin) and the lincosamide group (clindamycin). However, only a few MRSA strains proved resistant to streptomycin (12.5%) and ciprofloxacin (37.5%). β-lactam antibiotics, such as cefotaxime (100% resistant strains) and penicillin (58% resistant strains), were also ineffective against MSSA. Although MSSA isolates showed slight resistance to ticarcillin and erythromycin (33.3%) and clindamycin (25%), the remaining antibiotics proved effective (no resistant strains). **Conclusions:** Among the isolated strains, the greatest resistance to β-lactam antibiotics and erythromycin was observed. Multidrug-resistant strains of *S. aureus* were found in the emergency medical system. Even the MSSA strains detected in the studied ambulance showed resistance to some of the antibiotics used. The prevalence of *S. aureus* strains within ambulances indicates the need for a high hygiene level in daily prehospital work with patients.

## 1. Introduction

Methicillin-resistant *Staphylococcus aureus* (MRSA) strains have developed resistance to β-lactam antibiotics as a result of mutations in the *penicillin-binding protein* (*PB2a*). MRSA strains have been described as one of the leading causes of infections in patients and medical personnel in hospitals (HA-MRSA; hospital-associated MRSA) and in prehospital settings (CA-MRSA; community-associated MRSA) [1]. In Poland, approximately 15.5% of the population falls ill every year as a result of MRSA [2]. The problem of infections caused by the *S. aureus* strain presents an international epidemiological challenge [3].

The primary method of fighting infections caused by *S. aureus* is antibiotic therapy. Unfortunately, an increasing number of strains are showing resistance to antibiotics routinely used for treatment. The characteristics of the microorganism *S. aureus* contribute significantly to the severity of infections caused by it. As a result, *S. aureus* is considered a bacterium that leads to serious infections in humans, with subsequent health consequences [4]. Other strains of *Staphylococcus aureus* are called MSSA (methicillin-sensitive *Staphylococcus aureus*) and show sensitivity to β-lactam antibiotics. MSSA and MRSA strains are similar, but since the treatment of infections caused by methicillin-resistant *Staphylococcus aureus* is much more difficult, in such cases, vancomycin and linezolid therapy should be considered. The most important virulence factors in MRSA include production of PVL (Panton–Valentine leukocidin). Its action is to induce the lysis of leukocytes, mainly neutrophils, which leads, among other things, to tissue necrosis [5], and TSST-1 (toxic shock syndrome toxin 1), which is an exotoxin associated with toxic shock syndrome and has the ability to induce an excessive inflammatory response in the host body [6]. PSMs (phenol-soluble modulins) are another important virulence factor, which exhibit cytolytic activity against neutrophils and erythrocytes and have the ability to destroy cell membranes, which affects the immune system’s ability to fight infection [7], as well as haemolysins that cause complete or partial breakdown of red blood cells. Furthermore, alpha toxin has the ability to damage cells by creating pores in their cell membranes, resulting in leakage of cellular components and cell death [8]. The ability to form a biofilm is also extremely important since it provides bacteria with protection against the host’s immune response and against antibiotics by adhering to various surfaces [9]. Knowing the origins and modes of MRSA transmission is important for health care. It is also important to develop strategies to prevent and control the spread of the bacterial strains in question, as well as to implement appropriate treatments, including antibiotic therapy. Developing new effective medications and therapies and educating people about safe behaviours and precautions are also essential [10].

MRSA is generally transmitted through direct contact with infected persons or carriers. Bacterial transmission occurs by touching infected areas of the skin or contact with the patient’s bodily fluids. Another possibility is indirect contact with the contaminated area. MRSA is adapted to reside on various surfaces, such as medical equipment [6]. Medical procedures also constitute risk factors associated with MRSA transmission. Patients undergoing invasive procedures, e.g., bladder catheterisation or intubation [11], which are performed in the emergency room, are particularly exposed to these transmission routes. Another risk factor is the weakened immunity of patients, specifically those suffering from chronic diseases such as cardiovascular disease, alcohol addiction, neoplastic diseases, HIV (human immunodeficiency virus), or those on long-term antibiotic therapy [9].

MRSA can produce biofilm on a variety of abiotic and biotic surfaces, such as catheter tubes, endoprostheses, surgical instruments, and skin surfaces [12]. Biofilm also allows bacteria to survive in harsh environmental conditions, such as insufficient nutrient availability or changes in humidity [13]. Additionally, biofilm is often associated with chronic infections because, as mentioned, bacterial cells encapsulated in biofilm are less susceptible to the immune system and antibiotics [14].

The ambulance environment is a key area where both the patient and the staff are in a small space. Life-threatening conditions often require immediate procedures, which are carried out in a non-sterile environment. Open wounds, instrumented airway obstruction, blood vessels, pleural drainage, and intravenous and interosseous drug administration—these are just some procedures that require maximum asepticity, which cannot be fully ensured inside an ambulance, for obvious reasons [15,16,17,18]. This raises the question: Does providing assistance and transporting patients by emergency medical teams (EMTs) pose an additional risk due to secondary infection? The authors of the study assessed the presence of MRSA and MSSA strains in an emergency ambulance. A phenotypic evaluation of samples collected from an ambulance on permanent duty in the emergency medical system was conducted, and the drug resistance of the isolated strains was assessed.

## 2. Materials and Methods

### 2.1. Bacterial Strains

Specimens for analysis were collected using swabs with Amies transport medium after wetting the swab tip in RF solution (0.85% sterile saline). Ambulance disinfection is performed daily, and also immediately after any contamination occurs. Samples were taken before daily disinfection. In the second stage, the collected specimens were placed in BHI (brain heart infusion) broth (Graso, Starogard Gdański, Poland), which was incubated at 35 ± 1 °C for 18 ± 2 h. The affiliation of the isolates to the *S. aureus* species was confirmed by evaluating growth on nutrient agar supplemented with 5% sheep blood and on Chapman medium. Cell morphology and the ability to produce catalase were evaluated, and the presence of CF (clumping factor) on the cell surface was verified. The isolated bacteria, which degraded mannitol on Chapman medium, caused β-type haemolysis on nutrient agar supplemented with 5% sheep’s blood and were Gram-positive cocci in Gram-stained preparations and preliminarily identified as *S. aureus*. The presence of *S. aureus* was confirmed by a positive catalase production test and a positive CF result. The ATCC-positive control reference strain was not used.

### 2.2. Clumping Factor (CF) Test

In order to determine the affiliation of the isolates to the *S. aureus* species, a test was performed to assess the presence of the enzyme responsible for the conversion of fibrinogen into fibrin on bacterial cell surfaces. The tests used 18 ± 2 h cultures, which were conducted at 35 ± 1 °C in a BHI liquid medium (Graso, Poland). A drop of 0.85% NaCl was applied to a microscope slide. Bacterial cells were resuspended in a drop using an inoculation loop; then, a drop of rabbit plasma (Biomed, Kraków, Poland) was added and mixed by gently rocking the slide for a few seconds. The appearance of clear aggregates (flocs) in a droplet containing plasma indicated the presence of coagulase enzyme on the cell surface. The absence of aggregates in the control sample with a suspension of bacteria in a 0.85% NaCl solution indicated a negative test result.

### 2.3. Catalase Test

The isolated bacteria were evaluated for their ability to produce catalase. In order to assess this, a drop of 3% hydrogen peroxide solution (Aflofarm, Pabianice, Poland) was added to a bacterial culture incubated at 35 ± 1 °C on a solid medium. The observed intense decomposition of hydrogen peroxide into water and oxygen (developing gas bubbles) was indicative of catalase production and ruled out the presence of *Streptococcus* genus [19].

### 2.4. Gram Staining

Microscopic slides were prepared using Gram staining to assess cell morphology. The staining was performed on a degreased microscopic slide, onto which a drop of distilled water was applied, and a smear of the culture taken from Chapman medium (BTL, Warszawa, Poland) was made using an inoculation loop. The smear slide was allowed to dry, and then the slide was fixed in a torch flame. After fixing the slide, a solution of crystal violet was applied to it for two minutes, and excess dye was removed after some time elapsed. The preparation was treated with Lugol’s iodine for one minute, after which the entirety was decolorised with ethanol, the preparation was rinsed with water, and safranin was applied for 30 s. Finally, the entire preparation was rinsed and left to dry for evaluation under a microscope. Preparations that showed violet-stained cocci arranged in clusters were identified as Gram-positive.

### 2.5. Test of Strain Resistance to Selected Antibiotics

The antibiotic susceptibility profile of the strains was determined using the disc diffusion method following CLSI and EUCAST recommendations [20]. A 0.5 McFarland suspension (suspending colonies of a given strain in saline) was spread on Müeller–Hinton agar (BTL, Warszawa, Poland). MAST discs were then placed on the substrate 2 cm apart: E—erythromycin (15 μg), CIP—ciprofloxacin (5 μg), CD—clindamycin (2 μg), PG—penicillin (1 μg), TC—ticarcillin (75 μg), CTX—cefotaxime (5 μg), S—streptomycin (10 μg), and FOX—cefoxitin (30 μg). Samples made in this way were incubated at 35 ± 1 °C for 18 ± 2 h. Cefoxitin (30 μg) discs were used to confirm the bacteria’s affiliation with MRSA [21]. Strains that showed resistance to this antibiotic were classified as methicillin resistant.

## 3. Results

Swabs were taken, yielding 20 strains of *S. aureus*. Classification into species was based on bacterial growth on Chapman medium and microscope slides on which Gram-positive cocci were observed. A total of 39 swabs taken from the inside of the ambulance (13 sites) were analysed in 3 replicates, and *S. aureus* was found in 51% of the samples evaluated. Next, a bacterial resistance test to selected antibiotics was performed to determine the staphylococci affiliation to MRSA or MSSA strains. The test was conducted using cefoxitin discs and showed 8 strains resistant (40%) and 12 strains sensitive (60%) to this antibiotic. Sites where the highest incidence of MRSA was found included the tourniquet, pulse oximeter, stethoscope, glucometer, defibrillator (panels with buttons), and handles used to open and close doors. On the other hand, MSSA strains were isolated from the pressure stasis, blood pressure cuff, steering wheel, ECG cables, defibrillator (screen and panel with buttons), and stretcher at certain sites (head support, mattress). The results are presented in Table 1.

### 3.1. Evaluation of Growth on Chapman Substrate

#### Evaluation of the CF Tests, Catalase Tests, and Gram-Stained Slides

**Figure 1:**
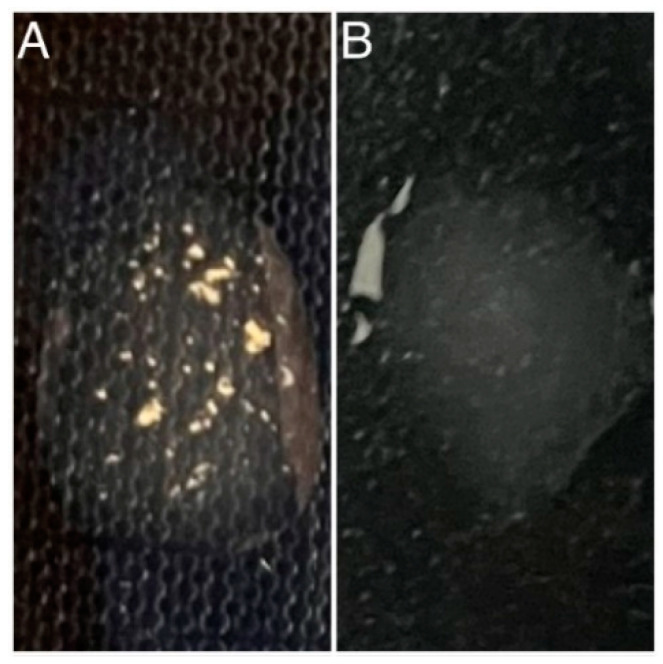
CF test. Positive result (**A**); negative result (**B**).

**Figure 2:**
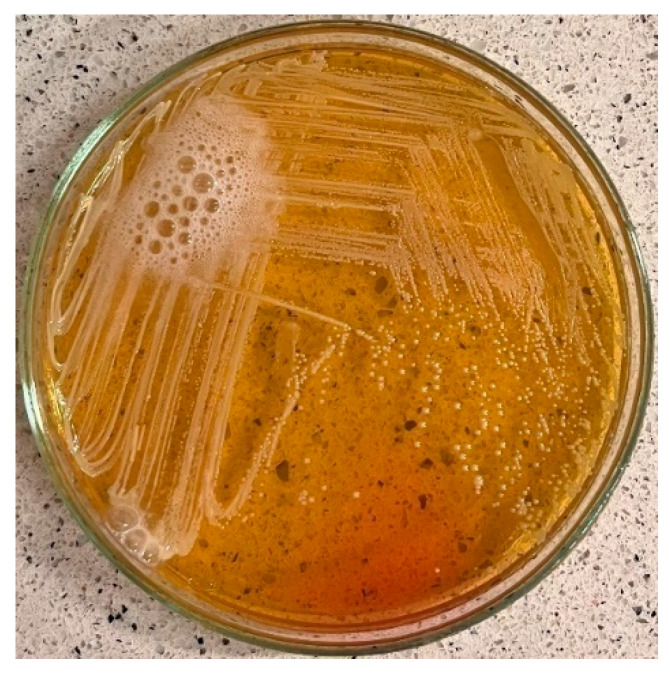
Positive catalase test result.

**Figure 3:**
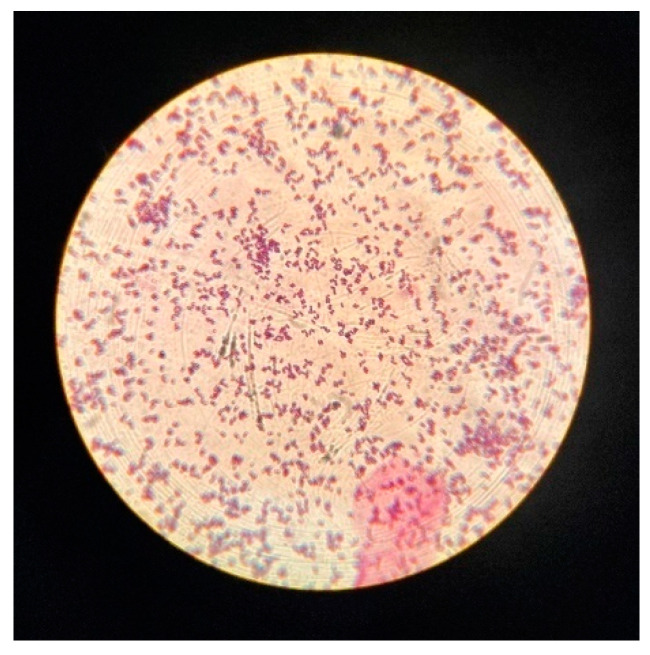
Gram-stained Gram-positive cocci.

The fibrinogen receptor test is routinely used to distinguish *S. aureus* from other staphylococcal species. Although most *S. aureus* strains are coagulase-positive (Figure 1), some are atypical and do not produce coagulase [22]. The analysis of CF test results revealed that 3 out of 20 isolates produced unexpected negative results (Figure 1) despite exhibiting mannitol fermentation, positive catalase, β-haemolysis, and the presence of Gram-positive cocci confirmed by Gram staining. The inability to produce coagulase may suggest a modified phenotype of this bacterium, potentially due to genetic mutations, environmental factors, or antibiotic exposure [23]. Therefore, in identifying species identity, it is crucial to interpret all tests and their results collectively. A positive result of the catalase test was observed in the case of all 20 strains analysed (Figure 2), and after Gram staining, the presence of Gram-positive cocci was observed in each case (Figure 3).

### 3.2. Evaluation of Haemolytic Properties

**Figure 4:**
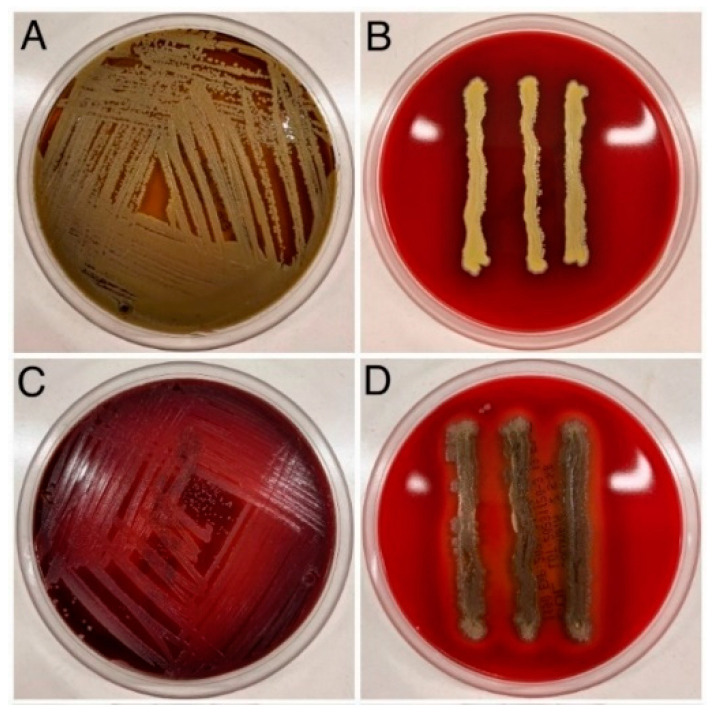
Haemolysis of the strains analysed. α-haemolysis (same strain in two replicates) (**A**,**B**); β-haemolysis (**C**,**D**).

Colonies growing on nutrient agar supplemented with 5% sheep blood (Graso, Poland) were analysed for morphology and haemolytic capacity. A total of 20 *S. aureus* strains were evaluated, of which 19 showed beta haemolysis, and 1 strain showed alpha haemolysis. These strains caused complete haemolysis of red blood cells and a visible zone of clarity around the bacterial colony on blood agar [24]. One strain showed alpha haemolysis (partially disintegrated red blood cells), leading to a green area around the bacterial colony on blood agar [25] (Figure 4). Gamma-type haemolysis was not observed. *Staphylococcus aureus* differs from other staphylococcal species in its β-haemolysis ability (in the vast majority of cases). This test can quickly determine the strain’s affiliation to the *S. aureus* species [26].

### 3.3. Antibiotic Resistance Evaluation of Tested Strains

All isolated *S. aureus* strains were tested for antibiotic susceptibility. A disc diffusion test for antibiotics used to treat infections caused by the bacteria in question was used for this purpose. As mentioned, a cefoxitin disc (30 μg) was used to identify MRSA strains [21]. A zone of inhibition smaller than 22 mm or the presence of single bacterial colonies in the zone of bacterial inhibition indicated MRSA according to the CLSI guidelines.

The antimicrobial resistance patterns of MRSA and MSSA isolates are summarised in Table 2. Almost 100% of MRSA isolates were resistant to antibiotics from the β-lactam group (penicillin, ticarcillin, cefotaxime, and cefoxitin), the macrolide group (erythromycin), and the lincosamide group (clindamycin). In contrast, only a few MRSA strains proved resistant to streptomycin (12.5%) and ciprofloxacin (37.5%). β-lactam antibiotics, such as cefotaxime (100% resistant strains) and penicillin (58% resistant strains), also proved ineffective against MSSA. While MSSA isolates showed slight resistance to ticarcillin and erythromycin (33.3%) and to clindamycin (25%), the remaining antibiotics proved effective (no resistant strains).

## 4. Discussion

This study confirmed the potential threat of MRSA strain transmission to emergency medical team personnel, as well as to patients. In Poland, 1718 strains of *S. aureus* were isolated in 2021 alone, 16.5% of which were MRSA [2]. Thirty-nine swabs were taken from inside the ambulance, from areas directly touched by medical personnel and patients. *S. aureus* strains were isolated from 51.28% of the samples, of which MRSA accounted for 40%. A study by Roline et al. [27] conducted in the United States showed that 47.6% of the ambulances in the group studied by these researchers tested positive for MRSA, while El-Mokhtar et al. [28] found 64.3% of *S. aureus* strains in medical vehicles in Egypt, of which 46.1% were MRSA. Bacterial contamination of ambulance surfaces and equipment is a serious problem worldwide, as it leads to the transmission of pathogens.

The sites where MRSA was found to be the most prevalent in the current study included: stasis (a band for drawing blood), pulse oximeter, stethoscope, glucometer, defibrillator (panels with buttons), and handles used to open and close doors. MSSA strains were isolated from the stasis (band for drawing blood), blood pressure cuff, steering wheel, ECG cables, defibrillator (screen and panel with buttons), and stretcher in certain spots (head support, mattress). The sites were selected due to frequent contact with medical personnel, patients, and bodily secretions from the subjects. The large number of *S. aureus* strains isolated in this study would seem to indicate that the disinfection used in the vehicle is insufficient, but in the case of the bacterium in question, such a statement would be inappropriate. One of the main virulence factors specific to *S. aureus* is biofilm formation. This structure effectively allows bacteria to survive on a variety of surfaces, including those being disinfected [29]. Some occupational settings tend to have higher S. aure-us colonisation rates (up to 80%). These include healthcare employees [30].

Due to the type of work they perform, paramedics are directly exposed to such infections, as well as pathogenic organisms and resistant organisms. This is mainly due to the lack of full knowledge of the patient’s condition. Paramedics work in medical emergencies, being forced to react quickly and act to save lives. Acting under time pressure, they are not always able to gather complete information about the patient’s condition. Furthermore, patients themselves are generally unaware of their carrier state. The data presented here confirm that emergency medical team personnel, due to their daily contact with medical vehicles (just like public transport), are particularly vulnerable to MRSA transmission [31].

The antibiotic resistance analyses of the isolated bacteria performed in this study showed that the tested *S. aureus* strains exhibited high resistance to selected antibiotics, such as cefotaxime (100%), penicillin G (70%), and erythromycin and ticarcillin (55% each). However, low resistance levels were observed for ciprofloxacin (15%) and streptomycin (5%). In contrast, El-Mokhtar et al. (2018) observed low resistance to cefotaxime (42.9%) and much higher resistance to ciprofloxacin (72.8%) [28]. The differences in antibiotic resistance results between the current analysis and the study conducted by Mokhtar et al. may be attributed to a number of factors. These factors could include geographic variations in the prevalence of bacterial strains and differences in antibiotic therapy practices in specific regions.

The detection of the presence of *S. aureus*, particularly MRSA strains, should be of concern because these bacteria can cause life-threatening diseases [32]. In the current study, the sites from which the strains in question were isolated were not surprising because this study was focused on equipment that emergency responders use in contact with patients [33]. Analyses of various microbial species present in ambulances are critical for controlling infections within different communities and hospital ranges.

Prevention methods such as hand hygiene, the use of personal protective equipment, the application of isolation procedures to patients, and recognition and control of infections have a significant impact on the transmission among humans. In the context of MRSA and MSSA infections, paramedics must be adequately trained in risk factors, modes of transmission, and identification of infection signs. This study showed that despite standard disinfection methods (surface and spray disinfectants), the vehicle contained dangerous strains of bacteria. Rescuers disinfect the contaminated surfaces after each patient, spraying a cleaning agent and wiping. Mist sprayers are also used to decontaminate the vehicle. However, the authors of this study did not intend to assess the quality of the disinfectants used. According to the guidelines for the prevention of transmission of biological pathogens of particular virulence or resistance, the control and prevention strategies include the following:

1. Performing a nasal swab screening test on a person suspected of being infected with or carrying *S. aureus*. According to the Polish Regulation of the Minister of Health of 7 March 2024, Section 30, paramedics have the ability to collect upper respiratory tract specimens from a patient (this gives medical personnel the ability to identify a person requiring isolation).
2. Hand hygiene. Evaluation of the effectiveness of hand washing procedures and the use of antiseptics by paramedics according to the 2009 WHO guidelines. The procedure recommends hand hygiene using an alcohol-based preparation in eight steps, for 20–30 s, and emphasises the use of gloves, which should be used for one patient. This point also includes the supply of hand sanitising agents to medical personnel in the patient area not more than 1–1.5 m away from the patient.
3. Isolation of the infected patient. When an infection is suspected or confirmed by screening test, paramedics should be trained in the proper use of precautions such as contact isolation to prevent the spread of bacteria. Additionally, it may be helpful to introduce disposable patient bedding/blankets into ambulances, which can be disposed of or sterilised after use. This can help reduce the spread of microorganisms around the ambulance.
4. Assessment of the availability and use of appropriate disinfectants and cleaning procedures for medical equipment and surfaces to prevent bacterial spread.
5. Evaluation of the effectiveness of procedures to monitor and report *S. aureus* infection cases in a medical facility and the effectiveness of actions taken in response to these reports. Identification of an infected patient who was transported to the hospital by ambulance should result in notifying the emergency medical service dispatcher about such an event in order to perform additional decontamination of the ambulance and the team.
6. Analysis of data on changes in the incidence of *S aureus* infections over time allows for the effectiveness of prevention and control strategies to be assessed and areas for improvement identified.
7. Public education. Increasing patient awareness about the routes of transmission and disease caused by *Staphylococcus aureus* can help reduce the risk of infection by promoting proper hand hygiene, using appropriate personal protective equipment, and encouraging the reporting of any signs of infection to medical personnel [34].

### Limitations of the Study

The authors of this study were limited in their testing for *S. aureus* strains. No cultures were performed for other types of bacteria (e.g., the *Enterobacteriaceae* family) that also pose a risk to humans. Based on their own experience working in the emergency medical service, the authors independently identified the sites of swabbing in the ambulance. Performing more tests on medical surfaces and materials could identify additional potential sites that create a transmission risk for the bacteria in question. There is very limited information in the literature regarding tests conducted inside an ambulance, which created the need to conduct the analysis according to the authors’ subjective assessment. Furthermore, this study was conducted inside an ambulance. Due to the specific environment, which was an ambulance constantly on duty, the possibility of duplicating the study in other vehicles was limited for organisational reasons.

## 5. Conclusions

Multidrug-resistant *S. aureus* strains, including MRSA strains, were found in an ambulance operating as an emergency medical service unit. The prevalence of *S. aureus* strains within ambulances indicates the need for a high hygiene level in daily prehospital work with patients. Among the strains studied, the greatest resistance was observed against β-lactam antibiotics. High resistance was also observed regarding erythromycin, which belongs to the macrolide group. Improving public awareness and education of *S. aureus* can help reduce the number of infections, thus reducing the burden on the healthcare system and improving the quality of patient care. It seems necessary to implement additional procedures in emergency ambulances, such as disposable sheets/blankets for patients, and exchange information with hospitals that have detected infectious material in a patient.

## Figures and Tables

**Table 1:** The results of the tests performed.

No.	Collection Site	Chapman	Coagulase	Catalase	Gram	Haemolysis	FOX
1.	Stasis (plastic part)	+	−	+	Z+	Β	S
2.	Stasis (material part)	+	+	+	Z+	Β	R
3.	SpO_2_ sensor	+	+	+	Z+	Β	R
4.	Pulse oximeter case	+	+	+	Z+	Β	R
5.	Pressure cuff	+	+	+	Z+	Β	S
6.	Stethoscope head	+	+	+	Z+	Β	R
7.	Stethoscope cable	+	−	+	Z+	Β	R
8.	Steering wheel—spot 1	+	+	+	Z+	Β	S
9.	Steering wheel—spot 2	+	+	+	Z+	Β	S
10.	Steering wheel—spot 3	+	+	+	Z+	Β	S
11.	ECG cables	+	+	+	Z+	Β	S
12.	Glucometer	+	+	+	Z+	Β	R
13.	Defibrillator right panel	+	+	+	Z+	Β	R
14.	Defibrillator left panel	+	−	+	Z+	Β	S
15.	Defibrillator screen	+	+	+	Z+	Β	S
16.	Stretcher (head support)—spot 1	+	+	+	Z+	Β	S
17.	Stretcher (torso)—spot 2	+	+	+	Z+	Β	S
18.	Stretcher (torso)—spot 3	+	+	+	Z+	Β	S
19.	Stretcher (legs)—spot 4	+	+	+	Z+	A	S
20.	Door handle (med. compartment)	+	+	+	Z+	Β	R

“+”—positive result; “−”—negative result; “Z+”—positive cocci; “B”—beta haemolysis; “A”—alpha haemolysis; “FOX”—cefoxitin; “S”—sensitive; “R”—resistant.

**Table 2:** Results of antibiotic susceptibility test readings of MRSA and MSSA strains.

Antibiotics Used [μg]	MRSA (n = 8)		MSSA (n = 12)		Sensitivity of Strains Isolated [%]	Resistance of Strains Isolated [%]
	Resistant	Sensitive	Resistant	Sensitive		
Penicillin (PG 1)	7 (87.5%)	1 (12.5%)	7 (58.33%)	5 (41.66%)	30	70
Ticarcillin (TC 75)	7 (87.5%)	1 (12.5%)	4 (33.33%)	8 (66.66%)	45	55
Cefoxitin (FOX 30)	8 (100%)	0	0	12 (100%)	60	40
Cefotaxime (CTX 5)	8 (100%)	0	12 (100%)	0	0	100
Streptomycin (S 10)	1 (12.5%)	7 (87.5%)	0	12 (100%)	95	5
Ciprofloxacin (CIP 5)	3 (37.5%)	5 (62.5%)	0	12 (100%)	85	15
Clindamycin (CD 2)	6 (75%)	2 (25%)	3 (25%)	9 (75%)	55	45
Erythromycin (E 15)	7 (87.5%)	1 (12.5%)	4 (33.33%)	8 (66.66%)	45	55

n—number of strains tested; μg—micrograms.

## Data Availability

The datasets generated and analysed during the current study are available from the corresponding author upon reasonable request.
